# Incentive Mechanisms for Carbon Emission Abatement Considering Consumers’ Low-Carbon Awareness under Cap-and-Trade Regulation

**DOI:** 10.3390/ijerph19074104

**Published:** 2022-03-30

**Authors:** Kelei Xue, Guohua Sun, Tongtong Yao

**Affiliations:** School of Management Science and Engineering, Shandong University of Finance and Economics, Jinan 250014, China; xuekelei@sdufe.edu.cn (K.X.); yaotongtong@mail.sdufe.edu.cn (T.Y.)

**Keywords:** low-carbon supply chain, incentive mechanism, carbon emission abatement, consumers low-carbon awareness, eco-social welfare, cap-and-trade

## Abstract

In the era of sustainable development, reducing carbon emissions and achieving carbon neutrality are gradually becoming a consensus for our society. This study explores firms’ incentive mechanisms for carbon emission abatement in a two-echelon supply chain under cap-and-trade regulation, where consumers exhibit low-carbon awareness. To boost the manufacturer’s motivation for abatement, the retailer can provide four incentive strategies, i.e., price-only (PO), cost-sharing (CS), revenue-sharing (RS), and both (cost and revenue) sharing (BS). The equilibrium decisions under the four incentive strategies are obtained by establishing and solving game models. A two-part tariff contract is also proposed to coordinate the low-carbon supply chain. Finally, through comparisons and analyses, we find that: (1) Consumers’ high low-carbon awareness can boost the manufacturer’s incentive for carbon emission abatement (CEA), thus increasing supply chain members’ profits. (2) It is more effective for the retailer to share its revenue to incentivize the manufacturer for abatement than to bear the investment cost of CEA. Thus, Strategy RS is better than Strategy CS and equivalent to Strategy BS. (3) The manufacturer and retailer have consistent incentive strategy preference under cap-and-trade regulation. Both firms prefer the incentive strategy with a higher cooperation level. (4) The incentive strategy with a higher cooperation level can also bring higher eco-social welfare under certain conditions.

## 1. Introduction

Sustainable development has received increasing attention in recent years, driven by climate changes, environmental pressures, regulations, and social responsibilities [1,2,3]. Many countries and regions have announced their low-carbon development strategies to achieve sustainable development. For example, Germany, the European Union (EU)’s largest emitter of carbon dioxide, has pledged to become carbon neutral by 2045 [4]. USA President Joe Biden has signed an executive order requiring the US federal government to become carbon neutral by 2050 [5]. China has also committed to peak carbon dioxide emissions by 2030 and carbon neutrality by 2060 [6]. Many national governments have also proposed various regulations, e.g., cap-and-trade mechanisms, to achieve these emissions reduction targets [7]. Under the cap-and-trade rule, the government assigns a carbon emission cap for a firm, and the firm can sell redundant or buy extra emission permits on the carbon trading market. Many countries, such as the EU and China, have established their own carbon trading markets to curb carbon emissions [8,9].

Consumers have an increasing sense of social responsibility in the carbon neutrality era, which has an important impact on sustainable supply chain operations. A global survey conducted by the Carbon Trust in 2020 indicates that 23% of customers consider the carbon emissions of products when making purchases [10]. Commercial research reveals that more than 40% of consumers are willing to pay more for green products [11]. A report by Chitra shows that consumers who exhibit a higher environmental awareness are more inclined to pay higher prices for eco-friendly products [12]. Consumers’ low-carbon awareness is a critical market driver that facilitates the manufacturer to develop carbon emission abatement (CEA) technology and produce decarbonized products [13]. In the apparel industry, H&M and Uniqlo have both invested in CEA technology to curb carbon emissions in production processes [14]. However, investing in CEA technology is a considerable expense for the manufacturer. Consumers’ willingness to buy low-carbon products can boost market demand, which has enormous profit potential for the downstream retailer. Intuitively, the downstream retailer has the motivation to help its upstream manufacturer invest in CEA technology. Therefore, it is necessary to study the firm’s incentive mechanisms for emissions abatement from a supply chain perspective.

The practical trend has witnessed that with increasing consumer low-carbon awareness, the retailer could cooperate with its manufacturer to reduce its products’ carbon levels. To achieve such cooperation, the firms employ various incentive strategies (e.g., revenue-sharing and cost-sharing) in business practice. For example, the Chinese giant retailer Suning cooperates with its upstream manufacturers, e.g., ANGEL, to improve the green product level by supporting green product design [15]. Another example comes from the case of Alpha Labs and Mega Pharmaceuticals. On the development of innovative diabetes drugs, Alpha and Mega reached an agreement in which they agreed to share equally in the development investment, e.g., 30% of U.S. domestic revenues and 80% of international revenues went to Mega, and the remainder accrued to Alpha [16]. Although scholars have widely discussed the role of cooperation strategies such as revenue-sharing and cost-sharing, little attention has been paid to their impacts on the incentive for carbon emission abatement, considering consumers’ low-carbon awareness under cap-and-trade regulation. Based on the above analysis, we propose the following research questions:

**Research Question 1:** What are the impacts of different incentive strategies (price-only, cost-sharing, revenue-sharing, and both-sharing) on the manufacturer’s equilibrium abatement decision and both firms’ profitability?

**Research Question 2:** What’s the equilibrium incentive strategy preference for the manufacturer and retailer with/without channel coordination? Are the supply chain members’ motivations for carbon emission abatement aligned?

**Research Question 3:** What are the environmental and social impacts of the different incentive strategies?

To answer the above research questions, we explore the manufacturer’s incentive mechanisms for carbon emission abatement in a two-echelon supply chain under cap-and-trade regulation where consumers exhibit low-carbon awareness and market demand depends on the product’s low-carbon level. To boost the manufacturer’s motivation for abatement, the retailer can provide four incentive strategies for him, i.e., price-only, cost-sharing, revenue-sharing, and both-sharing strategies. The equilibrium decisions under four incentive strategies are obtained by establishing and solving game models. Furthermore, a two-part tariff contract is also proposed to coordinate the low-carbon supply chain. We obtain many managerial insights through theoretical analysis, which can provide important decision-making references by which firms can establish appropriate cooperation mechanisms among supply chains to promote sustainable development.

The remainder of the paper is organized as follows. In Section 2, the related literature is reviewed. Section 3 describes the problem and model in detail. Then, we give four game models and corresponding equilibrium solutions in Section 4. Section 5 presents the full channel coordination. In Section 6, we compare and analyze the following equilibrium results. Concluding remarks and some directions for future research are provided in Section 7. Finally, all proofs are presented in Appendix A to make the paper more readable.

## 2. Literature Review

Our paper is related to two categories of literature: low-carbon supply chains, and cooperation and coordination in supply chains. In this section, we will review each category of literature and highlight the differences between the relevant research and our work.

### 2.1. Low-Carbon Supply Chains

In recent years, many scholars have studied various topics in a low-carbon supply chain. For example, some scholars have studied the supply chain’s green/low-carbon technology investment issue. Xue et al. [17] studied green product design strategies under different supply chain structures and government subsidy strategies. Dong et al. [18] examined who should lead the investment in green product development in a supply chain. Xia et al. [19] studied how reciprocal awareness and consumers’ low-carbon awareness affect the decisions of carbon emission reduction and the pricing and performances of the supply chain members under the cap-and-trade policy. Qi et al. [20] investigated joint decisions on emission reduction investment and order quantity under the conditional value at risk framework. Qin et al. [21] explored the carbon emission reduction and financing strategies of capital-constrained manufacturers under the cap-and-trade scheme. Xu and Duan [22] examined the ‘greenness’ investment and pricing strategies with government subsidies and explored when to adopt blockchain technology. Yang et al. [7] explored the manufacturer’s joint decisions of carbon emission reduction and channel selections under cap-and-trade regulation.

Some other papers examine the various carbon policies used to control and curb carbon emissions. Benjaafar et al. [23] studied the production issues under carbon offsets, carbon tax, cap-and-trade, and strict carbon caps policies. Li et al. [24] explored the impacts of absolute-cap and intensity-cap carbon regulations on supply chain decisions with carbon reduction efforts. Based on an economic order quantity model, He et al. [25] examined the production lot-sizing problems under cap-and-trade and carbon tax regulations. Xu et al. [26] studied the joint production and pricing problem under cap-and-trade and carbon tax regulations. Chen et al. [27] investigated the optimal carbon tax design problem in a low-carbon supply chain. Fang et al. [28] studied the influence of carbon tariffs on global emission control in a global supply chain. Entezaminia et al. [29] addressed the joint production and carbon trading problems for unreliable manufacturing systems under cap-and-trade regulation.

Some researchers have also studied the interplay of operations, finance, and the environment in a low-carbon supply chain (e.g., [30,31,32,33]). For example, Zhao et al. [34] studied the call option contract in a low-carbon supply chain with a risk-averse retailer under carbon tax regulation. Wu et al. [35] determined the effects of carbon emissions reduction on supply chain operations and financing decisions. Cao et al. [36] explored the impact of investments in carbon abatement on the financing modes preference in an emission-dependent supply chain. Tang and Yang [37] investigated how the channel power structure influences the financing mechanism, carbon emissions abatement, and performance of the low-carbon supply chain. The government can also provide subsidies and green credit for carbon emission abatement. Li et al. [38] analyzed the impact of government subsidies on the low-carbon supply chain. An et al. [39] compared green credit financing with trade credit financing in a supply chain with carbon emission limits.

### 2.2. Cooperation and Coordination in Supply Chain

In a low-carbon supply chain, the firm’s investments to reduce carbon emissions are often expensive. In order to motivate the firm to invest, the other firms in the supply chain can adopt various cooperation strategies. Wang et al. [40] studied three cooperation scenarios: non-cooperation, a cooperation program, and a two-way cooperation contract. In each cooperation scenario, they developed differential game models by which to study the emission reduction decisions. Wang et al. [41] discussed the cost-sharing and wholesale price premium contracts. Their study examines the effects of the channel power structures on carbon emission reduction decisions. Sun and Li [42] proposed an optimization model for sharing emission reductions by integrating DEA and energy conservation and emission reduction (ECER) technology. Ghosh and Shah [43] studied two types of cost-sharing models: one in which the cost-sharing contract is offered by the retailer, and the other in which the cost-sharing contract is negotiated between the retailer and manufacturer. Hong and Guo [44] studied three cooperation contracts: price-only, green marketing cost-sharing, and two-part tariff contracts, within a green product supply chain, and explored their environmental impacts. Li et al. [45] examined the price-only, cost-sharing, and revenue-sharing strategies under contracting–designing and contracting–marketing formats, respectively.

Some studies also research pricing, carbon emission reduction, and coordination in a low-carbon supply chain. Bai et al. [8] examined the coordination problem of a low-carbon supply chain with two products under cap-and-trade regulation. Bai et al. [46] explored the impacts of the emissions reduction technology investment and risk aversion on the supply chain coordination under a carbon tax policy. Xu et al. [47] studied the coordination mechanism in a make-to-order supply chain. Xu et al. [48] revealed that wholesale price and cost-sharing contracts could coordinate a make-to-order supply chain with green technology. Yang and Chen [49] investigated the impact of revenue-sharing (RS) and cost-sharing (CS) schemes under a carbon tax policy and found that the RS scheme failed to coordinate the supply chain. Qian et al. [50] studied two non-cooperative and two cooperative contracts (Nash bargaining and Rubinstein bargaining) in a two-echelon sustainable supply chain considering a fair-minded retailer.

### 2.3. Summary of the Literature Review

In order to emphasize the novelty of our paper more clearly, we provide Table 1 to present the main differences between our work and related studies. The most relevant articles are Hong and Guo [44], Li et al. [45], and Yang and Chen [49]. In the study of Hong and Guo [44], the manufacturer provides cooperation contracts to the retailer. Additionally, the cost-sharing contract is about sharing the green marketing cost. However, in our paper, we examine the cooperation strategies offered by the retailer to the manufacturer. The retailer can share its sales revenue and investment cost of the carbon abatement technology with the manufacturer. Differently from Li et al.’s paper [45], they explored the effects of the marketing effort on the equilibrium decisions in a green supply chain. However, in our paper, we explore the impacts of consumers’ low-carbon awareness. Moreover, we also investigate the supply chain coordination issue and how the carbon policy affects the economy and environment. In a low-carbon supply chain, Yang and Chen [49] studied the carbon tax policy. Their paper does not consider the supply chain coordination and the impact of the other carbon policies. Unlike their study, our paper investigates the cooperation and coordination issues in a low-carbon supply chain considering consumers’ low-carbon awareness under cap-and-trade regulation. In conclusion, in our paper, we explore the price-only, cost-sharing, revenue-sharing, and both-sharing strategies provided by the retailer to the manufacturer in an emission-dependent supply chain under cap-and-trade regulation. We also propose a two-part tariff coordination contract and give two firms’ equilibrium incentive strategy preferences with/without channel coordination. At last, the environmental and social impacts of the four incentive strategies and coordination contract are also analyzed.

## 3. Model Descriptions

We consider one two-echelon supply chain consisting of one manufacturer (he) and one retailer (she). The manufacturer produces low-carbon products under cap-and-trade regulation. The retailer wholesales eco-friendly products from her manufacturers and sells them to consumers in the market. The manufacturer can invest in carbon emission abatement (CEA) technology to produce low-carbon products to appeal to consumers. The manufacturer’s investment cost of CEA technology can be expressed by a quadratic function, i.e., $C\left(\theta \right)=\frac{1}{2}k\theta^{2}$, where θ is the low-carbon products’ CEA level, and k is a cost coefficient. This quadratic cost function is widely used in the relevant literature, e.g., Xue et al. [17], Wei et al. [51]. The variable production cost of each low-carbon product is assumed to be c. Symbols and notations used in this paper are concluded in Table 2.

In this paper, we consider that consumers have low-carbon awareness and are more willing to purchase low-carbon products. Thus, the demand function can be expressed by

(1) q=a−p+λθ,

where a represents the primary market scale of the low-carbon products, and $\lambda \in \left(0,1\right)$ denotes the sensitivity of the market demand concerning the products’ CEA level. We use λ to measure the consumers’ low-carbon awareness (CLA) level. A higher CLA level means that the consumers are more inclined to pay higher prices for low-carbon products. This form of demand function is widely used in the relevant literature, such as in Tsay and Agrawal [38], Yang et al. [39], and Yang and Chen [49].

Under cap-and-trade regulation, the government gives the manufacturer a carbon quota (or cap) of G. The manufacturer can sell redundant or buy extra emission permits on the carbon trading market. Therefore, cap-and-trade regulation can be regarded as a financial incentive to encourage manufacturers to invest in CEA technology. Without the manufacturer’s CEA investment, the products’ initial carbon emission level is assumed to be e. The carbon trading amount of the manufacturer is:

(2) $$T=e\left(1-\theta \right)q-G,$$

where T>0 denotes the manufacturer’s need to buy extra carbon credits from the carbon trading market, and T<0 means that the manufacturer can sell redundant carbon credits on the carbon trading market. Moreover, we use h to represent the carbon trading price, which is an exogenous variable determined by the carbon trading market.

**Figure 1:**
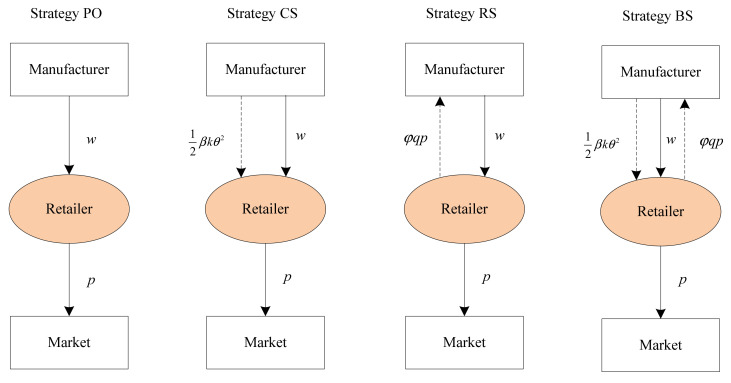
The retailer’s four incentive strategies.

To boost the manufacturer’s incentive for abatement, the retailer can provide four incentivizing strategies for him, i.e., sharing the sales revenue solely, sharing the investment cost of CEA technology solely, sharing both revenue and investment cost simultaneously, or sharing neither of the two with the manufacturer. In sequence, we denote the four incentive strategies as revenue-sharing, cost-sharing, both-sharing, and price-only strategies. Moreover, we use the symbol $\left\{\beta ,\;\varphi \right\}$ to indicate the retailer’s particular incentive strategy, where β and φ represent the proportion of investment cost of CEA technology and the sales revenue that she would share with her manufacturer, respectively. In the following, we will describe the four incentive strategies in detail (refer to Figure 1).

(1) **Price-only strategy** (Strategy PO): This strategy corresponds to the scenario where β=0 and φ=0. In this strategy, the retailer shares neither the investment cost of CEA technology nor her revenue with the manufacturer. The manufacturer and retailer make independent decisions to maximize their own profit.
(2) **Cost-sharing strategy** (Strategy CS): This strategy corresponds to the scenario where β>0 and φ=0. In this strategy, in order to stimulate the manufacturer to invest in developing low-carbon technology to produce low-carbon products, the retailer shares β proportion of the total investment cost of CEA technology.
(3) **Revenue-sharing strategy** (Strategy RS): This strategy corresponds to the scenario where β=0 and φ>0. Unlike Strategy CS, in this strategy, the downstream retailer shares $\varphi \in \left(0,1\right)$ proportion of her revenue with the upstream manufacturer.
(4) **Both-sharing strategy** (Strategy BS): This strategy corresponds to the scenario where β>0 and φ>0. Strategy BS is a combination of Strategy CS and Strategy RS. In the BS strategy, the retailer shares both the investment cost of CEA technology and her revenue with her upstream manufacturer.

Therefore, the manufacturer’s profit function under the incentive strategy $\left\{\beta ,\varphi \right\}$ is

(3) $$\pi_{m}\left(w,\theta \right)=\left(\varphi p+w-c\right)q-h\left(e\left(1-\theta \right)q-G\right)-\frac{1}{2}\left(1-\beta \right)k\theta^{2},$$

where the first term is the manufacturer’s revenue from selling low-carbon products to the retailer, the second term is the cost or income from carbon trading, and the third term is the investment cost of the manufacturer’s CEA technology. The retailer’s profit function under incentive strategy $\left\{\beta ,\varphi \right\}$ is:

(4) $$\pi_{r}\left(p\right)=\left(\left(1-\varphi \right)p-w\right)q-\frac{1}{2}\beta k\theta^{2},$$

The profit of the entire supply chain system under incentive strategy $\left\{\beta ,\varphi \right\}$ is:

(5) $$\pi_{sc}\left(p,\theta \right)=\left(p-c\right)q-h\left(e\left(1-\theta \right)q-G\right)-C\left(\theta \right).$$

## 4. Equilibrium Solutions

In this section, considering whether the retailer shares the investment cost of the CEA technology or her revenue, or both the investment cost and the revenue, or neither of the two, with the manufacturer, we model and explore the price-only, cost-sharing, revenue-sharing, and both-sharing strategies. The backward induction method is used to solve the above models. We assume that the conditions of $k>{\left(eh+\lambda \right)}^{2}$ and a>c+eh hold so that the equilibrium solutions exist in four models. Superscript “PO”, “CS”, “RS”, and “BS” are used to denote the corresponding variables under four different incentive strategies. We also use superscript “*” to mark the optimum value.

### 4.1. Price-Only Strategy

In this strategy, the manufacturer and retailer both decide to maximize their own profit under the Stackelberg game framework. The manufacturer, as the Stackelberg leader, maximizes his profit by optimally determining the wholesale price $w^{PO}$ and CEA level $\theta^{PO}$. As the Stackelberg follower, given the optimal decisions of the manufacturer, the retailer makes her optimal decision on retail price $p^{PO}$. Therefore, the Stackelberg game problem in Strategy PO can be formulated as:

(6) $$\left\{\begin{array}{c} \underset{w^{PO},\theta^{PO}}{max}\pi_{m}^{PO}\left(w^{PO},\theta^{PO}\right)=\left(w^{PO}-c\right)q^{PO}-h\left(e\left(1-\theta^{PO}\right)q^{PO}-G\right)-\frac{1}{2}k\theta^{PO}{}^{2}\\ p^{PO*}\;is\;derived\;from\;solving\;the\;following\;problem\\ \underset{p^{PO}}{max}\pi_{r}^{PO}\left(p^{PO}\right)=\left(p^{PO}-w^{PO}\right)q^{PO} \end{array}\right.,$$

where $q^{PO}$ is given by Equation (1). Solving the above problem, we can derive the following Theorem 1.

**Theorem** **1.** *In Strategy PO, the equilibrium wholesale pricing, retail pricing, order quantity, and CEA level of the low-carbon supply chain are given by:*

(7) $$w^{PO*}=\frac{a\left(2k-eh\left(eh+\lambda \right)\right)+\left(c+eh\right)\left(2k-\lambda \left(eh+\lambda \right)\right)}{4k-{\left(eh+\lambda \right)}^{2}},$$

(8) $$p^{PO*}=\frac{a\left(3k-eh\left(eh+\lambda \right)\right)+\left(c+eh\right)\left(k-\lambda \left(eh+\lambda \right)\right)}{4k-{\left(eh+\lambda \right)}^{2}},\;$$

(9) $$q^{PO*}=\frac{k\left(a-c-eh\right)}{4k-{\left(eh+\lambda \right)}^{2}},$$

*and*

(10) $$\theta^{PO*}=\frac{\left(a-c-eh\right)\left(eh+\lambda \right)}{4k-{\left(eh+\lambda \right)}^{2}}.$$

According to Theorem 1, the equilibrium decisions for the low-carbon supply chain exist in Strategy PO. Substituting the equilibrium solutions given by Theorem 1 into the profit functions of the manufacturer, retailer, and whole supply chain, we derive the following:

(11) $$\pi_{m}^{PO*}=Gh+\frac{k{\left(a-c-eh\right)}^{2}}{2\left(4k-{\left(eh+\lambda \right)}^{2}\right)},$$

(12) $$\pi_{r}^{PO*}=\frac{k^{2}{\left(a-c-eh\right)}^{2}}{{\left(4k-{\left(eh+\lambda \right)}^{2}\right)}^{2}},$$

(13) $$\pi_{sc}^{PO*}=Gh+\frac{k{\left(a-c-eh\right)}^{2}\left(6k-{\left(eh+\lambda \right)}^{2}\right)}{2{\left(4k-{\left(eh+\lambda \right)}^{2}\right)}^{2}}.$$

In the light of the above theorem, the carbon quota given by the government has no impact on the equilibrium decisions of the low-carbon supply chain. However, a larger carbon quota can bring more profit to the manufacturer. To explore the effects of the CLA level and CEA investment cost coefficient, Corollary 1 is given as follows.

 **Corollary** **1.**  *In Strategy PO, the equilibrium decisions and profits have the following properties:*

 *(i)* 
$\frac{\partial \theta^{PO*}}{\partial \lambda }>0,\;\frac{\partial q^{PO*}}{\partial \lambda }>0,\;\frac{\partial \pi_{m}^{PO*}}{\partial \lambda }>0,\;\frac{\partial \pi_{r}^{PO*}}{\partial \lambda }>0,\;\frac{\partial \pi_{sc}^{PO*}}{\partial \lambda }>0;$

 *(ii)*  $\frac{\partial \theta^{PO*}}{\partial k}<0,\;\frac{\partial q^{PO*}}{\partial k}<0,\;\frac{\partial \pi_{m}^{PO*}}{\partial k}<0,\;\frac{\partial \pi_{r}^{PO*}}{\partial k}<0,\;\frac{\partial \pi_{sc}^{PO*}}{\partial k}<0$.

Corollary 1(i) indicates that with the increase of the CLA level, both the manufacturer’s CEA level and the retailer’s order quantity increase, which also leads to the rise of their profit and total supply chain profit. This conclusion is intuitive. If consumers are sensitive to the CEA level of the products, the retailer will place a larger order with the manufacturer. The manufacturer also has enough incentive to invest in CEA technology. In such a circumstance, both manufacturer and retailer will benefit. This implies that cultivating consumers’ environmental awareness is beneficial to the sustainable development of the low-carbon supply chain from economic and ecological perspectives.

Corollary 1(ii) shows that the CEA level, manufacturer’s profit, order quantity, and the retailer’s profit all decrease in the CEA’s investment cost coefficient. This conclusion is also intuitive. If the CEA investment is expensive, the manufacturer is more inclined to buy extra carbon emission credits from the carbon trading market rather than invest in CEA technology. Lower investment in CEA technology leads to the supply chain members having lower order quantity and profits. This gives us a vital managerial implication that improving the CEA investment efficiency is conducive to the sustainable development of the low-carbon supply chain.

### 4.2. Cost-Sharing Strategy

In this strategy, the retailer shares $\beta^{CS}$ proportion of the CEA investment cost with the manufacturer, i.e., $\frac{1}{2}\beta^{CS}k\theta^{CS}{}^{2}$. The retailer first determines her optimal cost-sharing percentage $\beta^{CS}$. Then, the manufacturer determines the optimal wholesale price $w^{CS}$ and CEA level $\theta^{CS}$. Lastly, the retailer makes a decision on retail price $p^{CS}$ to maximize her profit. Therefore, the retailer-led cost-sharing game problem in Strategy CS is given by:

(14) $$\left\{\begin{array}{c} \underset{\beta^{CS}}{max}\pi_{r}^{CS}\left(\beta^{CS}\right)=\left(p^{CS*}-w^{CS*}\right)q^{CS}-\frac{1}{2}\beta^{CS}k\theta^{CS*}{}^{2}\\ w^{CS*}\;and\;\theta^{CS*}\;are\;derived\;from\;solving\;the\;following\;problem\\ \left\{\begin{array}{c} \underset{w^{CS},\theta^{CS}}{max}\pi_{m}^{CS}\left(w^{CS},\theta^{CS}\right)=\left(w^{CS}-c\right)q^{CS}-h\left(e\left(1-\theta^{CS}\right)q^{CS}-G\right)-\frac{1}{2}\left(1-\beta^{CS}\right)k\theta^{CS}{}^{2}\\ p^{CS*}\;is\;derived\;from\;solving\;the\;following\;problem\\ \underset{p^{CS}}{max}\pi_{r}^{CS}\left(p^{CS}\right)=\left(p^{CS}-w^{CS}\right)q^{CS}-\frac{1}{2}\beta^{CS}k\theta^{CS}{}^{2} \end{array}\right. \end{array}\right.$$

where $q^{CS}$ is given by Equation (1). Solving the above problem, we can derive the following Theorem 2.

**Theorem** **2.** *In Strategy CS, the equilibrium wholesale pricing, retail pricing, order quantity, cost-sharing percentage, and CEA level of the low-carbon supply chain are given by:*

(15) $$w^{CS*}=\frac{a\left(8k-\left(eh+\lambda \right)\left(5eh+\lambda \right)\right)+\left(c+eh\right)\left(8k-\left(eh+\lambda \right)\left(eh+5\lambda \right)\right)}{2\left(8k-3{\left(eh+\lambda \right)}^{2}\right)},$$

(16) $$p^{CS*}=\frac{a\left(24k-\left(eh+\lambda \right)\left(11eh+3\lambda \right)\right)+\left(c+eh\right)\left(8k-\left(eh+\lambda \right)\left(eh+9\lambda \right)\right)}{4\left(8k-3{\left(eh+\lambda \right)}^{2}\right)},\;$$

(17) $$q^{CS*}=\frac{\left(a-c-eh\right)\left(8k-{\left(eh+\lambda \right)}^{2}\right)}{4\left(8k-3{\left(eh+\lambda \right)}^{2}\right)},$$

(18) $$\beta^{CS*}=\frac{{\left(eh+\lambda \right)}^{2}}{8k},$$

*and*

(19) $$\theta^{CS*}=\frac{2\left(a-c-eh\right)\left(eh+\lambda \right)}{8k-3{\left(eh+\lambda \right)}^{2}}.$$

Similarly, there exist the equilibrium decisions for the low-carbon supply chain in Strategy CS. Substituting the equilibrium solutions given by Theorem 2 into the profit functions of the manufacturer, retailer, and whole supply chain, we derive the following:

(20) $$\pi_{m}^{CS*}=\frac{1}{24}\left(a^{2}-2aeh+24Gh+e^{2}h^{2}+\frac{16k{\left(a-eh\right)}^{2}}{8k-3{\left(eh+\lambda \right)}^{2}}\right),$$

(21) $$\pi_{r}^{CS*}=\frac{{\left(a-eh\right)}^{2}\left(8k+{\left(eh+\lambda \right)}^{2}\right)}{16\left(8k-3{\left(eh+\lambda \right)}^{2}\right)},$$

(22) $$\pi_{sc}^{CS*}=\frac{1}{48}\left(a^{2}-2aeh+48Gh+e^{2}h^{2}+\frac{64k{\left(a-eh\right)}^{2}}{8k-3{\left(eh+\lambda \right)}^{2}}\right).$$

The impact of carbon quota on the equilibrium solutions and profits in Strategy CS is similar to that in Strategy PO. In order to analyze how the CLA level and CEA investment cost coefficient affect the equilibrium decisions and profits of the low-carbon supply chain, Corollary 2 is given as follows.

 **Corollary** **2.**  *In Strategy CS, the equilibrium decisions and profits have the following properties:*

 *(i)* 
$\frac{\partial \beta^{CS*}}{\partial \lambda }>0,\;\frac{\partial \theta^{CS*}}{\partial \lambda }>0,\;\frac{\partial q^{CS*}}{\partial \lambda }>0,\;\frac{\partial \pi_{m}^{CS*}}{\partial \lambda }>0,\;\frac{\partial \pi_{r}^{CS*}}{\partial \lambda }>0,\;\frac{\partial \pi_{sc}^{CS*}}{\partial \lambda }>0;$

 *(ii)*  $\frac{\partial \beta^{CS*}}{\partial k}<0,$ $\frac{\partial \theta^{CS*}}{\partial k}<0,\;\frac{\partial q^{CS*}}{\partial k}<0,\;\frac{\partial \pi_{m}^{CS*}}{\partial k}<0,\;\frac{\partial \pi_{r}^{CS*}}{\partial k}<0,\;\frac{\partial \pi_{sc}^{CS*}}{\partial k}<0$.

According to Corollary 2, the retailer’s optimal cost-sharing percentage increases in the CLA level, while decreases in the investment cost coefficient, which shows that the retailer will share a more considerable proportion of CEA investment cost with her manufacturer if the consumers exhibit higher low-carbon awareness or if CEA investment is not so expensive. This conclusion is consistent with our intuition. The reason is that when the CLA level is higher or the investment cost coefficient is lower, it always means there is an enormous market profit potential. Therefore, the retailer will motivate the manufacturer to maximize her profit by sharing a larger proportion of the CEA investment cost. This conclusion is different from that of Hong and Guo [44]. In their study, the cost-sharing rate is a constant.

The CEA level and profit of the manufacturer, the order quantity, and the retailer’s profit increase the CLA level and decrease the investment cost coefficient of the CEA. The underlying managerial insights in Strategy CS are identical to those in Strategy PO, so we omit the details for brevity.

### 4.3. Revenue-Sharing Strategy

In this strategy, we establish a retailer-led revenue-sharing game model where the retailer shares φ proportion of her revenue with the manufacturer to stimulate the manufacturer to invest in low-carbon technology. That is to say, at the end of the selling season, the retailer will give the amount of income, $\varphi^{RS}p^{RS}q^{RS}$, to the manufacturer. Therefore, the retailer-led revenue-sharing game problem in Strategy RS can be given by:

(23) $$\left\{\begin{array}{c} \underset{\varphi^{RS}}{max}\pi_{r}^{RS}\left(\varphi^{RS}\right)=\left(\left(1-\varphi^{RS}\right)p^{RS*}-w^{RS*}\right)q^{RS}\\ w^{RS*}\;and\;\theta^{RS*}\;are\;derived\;from\;solving\;the\;following\;problem:\\ \left\{\begin{array}{c} \underset{w^{RS},\theta^{RS}}{max}\pi_{m}^{RS}\left(w^{RS},\theta^{RS}\right)=\left(\varphi^{RS}p^{RS*}+w^{RS}-c\right)q^{RS}-h\left(e\left(1-\theta^{RS}\right)q^{RS}-G\right)-\frac{1}{2}k\theta^{RS}{}^{2}\\ p^{RS*}\;is\;derived\;from\;solving\;the\;following\;problem:\\ \underset{p^{RS}}{max}\pi_{r}^{RS}\left(p^{RS}\right)=\left(\left(1-\varphi^{RS}\right)p^{RS}-w^{RS}\right)q^{RS} \end{array}\right. \end{array}\right.$$

where $q^{RS}$ is given by Equation (1). Solving the above problem, we can derive the following Theorem 3.

**Theorem** **3.** *In Strategy RS, the equilibrium wholesale pricing, retail pricing, order quantity, revenue-sharing percentage, and CEA level of the low-carbon supply chain are given by*:

(24) $$w^{RS*}=\frac{a\left(2k-\left(eh+\lambda \right)\left(2eh+\lambda \right)\right)+\left(c+eh\right)\left(2k-\lambda \left(eh+\lambda \right)\right)}{4k},$$

(25) $$p^{RS*}=\frac{a\left(3k-\left(eh+\lambda \right)\left(2eh+\lambda \right)\right)+\left(c+eh\right)\left(k-\lambda \left(eh+\lambda \right)\right)}{2\left(2k-{\left(eh+\lambda \right)}^{2}\right)},\;$$

(26) $$q^{RS*}=\frac{k\left(a-c-eh\right)}{2\left(2k-{\left(eh+\lambda \right)}^{2}\right)},$$

(27) $$\varphi^{RS*}=\frac{{\left(eh+\lambda \right)}^{2}}{2k},$$

*and*

(28) $$\theta^{RS*}=\frac{\left(a-c-eh\right)\left(eh+\lambda \right)}{2\left(2k-{\left(eh+\lambda \right)}^{2}\right)}.$$

Substituting the above equilibrium solutions given by Theorem 3 into Equation (23), the profits of the manufacturer, retailer, the total supply chain can be derived as follows:

(29) $$\pi_{m}^{RS*}=Gh+\frac{k{\left(a-c-eh\right)}^{2}}{4\left(2k-{\left(eh+\lambda \right)}^{2}\right)},$$

(30) $$\pi_{r}^{RS*}=\frac{k{\left(a-c-eh\right)}^{2}}{8\left(2k-{\left(eh+\lambda \right)}^{2}\right)},$$

(31) $$\pi_{sc}^{RS*}=Gh+\frac{3k{\left(a-c-eh\right)}^{2}}{8\left(2k-{\left(eh+\lambda \right)}^{2}\right)}.$$

The impact of carbon quota on the equilibrium solutions and profits in Strategy RS is similar to those in Strategies CS and PO. According to Theorem 3, we can easily derive the following Corollary 3:

**Corollary** **3.** *There exists the relationship of* $\varphi^{RS*}=4\beta^{CS*}.$

Corollary 3 shows that in Strategy RS, the retailer is willing to share a higher percentage, i.e., fourfold, of revenue relative to the percentage of CEA investment cost borne in Strategy CS, which is different from the conclusions of the related studies of Hong and Guo [44], and Li et al. [45]. This result implies that the cooperation level between the retailer and manufacturer in Strategy RS is higher than that in Strategy CS.

In order to analyze how the CLA level and CEA investment cost coefficient affect the equilibrium decisions and profits of the low-carbon supply chain, Corollary 4 is given as follows.

 **Corollary** **4.**  *In Strategy RS, the equilibrium decisions and profits have the following properties:*

 *(i)* 
$\frac{\partial \theta^{RS*}}{\partial \lambda }>0,\;\frac{\partial \varphi^{RS*}}{\partial \lambda }>0,\;\frac{\partial q^{RS*}}{\partial \lambda }>0,\;\frac{\partial \pi_{m}^{RS*}}{\partial \lambda }>0,\;\frac{\partial \pi_{r}^{RS*}}{\partial \lambda }>0,\;\frac{\partial \pi_{sc}^{RS*}}{\partial \lambda }>0;$

 *(ii)*  $\frac{\partial \theta^{RS*}}{\partial k}<0,\;\frac{\partial \varphi^{RS*}}{\partial k}<0,\;\frac{\partial q^{RS*}}{\partial k}<0,\;\frac{\partial \pi_{m}^{RS*}}{\partial k}<0,\;\frac{\partial \pi_{r}^{RS*}}{\partial k}<0,\;\frac{\partial \pi_{sc}^{RS*}}{\partial k}<0$.

In light of Corollary 4, the retailer’s optimal revenue-sharing percentage increases in CLA level, while decreases in the investment cost coefficient of CEA, which indicates that the retailer will share a more considerable proportion of her earning with the manufacturer if the consumers exhibit higher low-carbon awareness or if CEA investment is not too expensive. This conclusion is also consistent with our intuition. The reason is similar to that in Strategy CS. Similarly, the impacts of the CLA on the CEA level and ordering decisions and profits of the low-carbon supply chain are also identical to those in Strategies CS and PO. Thus, we omit them here.

### 4.4. Both-Sharing Strategy

In this strategy, we establish a retailer-led, both cost- and revenue-sharing game model in which the retailer shares both $\beta^{BS}$ proportion of the CEA investment cost and $\varphi^{BS}$ proportion of her revenue with the manufacturer. Firstly, the retailer decides her optimal cost-sharing percentage $\beta^{BS}$ and revenue-sharing percentage $\varphi^{BS}$. Secondly, the manufacturer determines the optimal wholesale price $w^{BS}$ and CEA level $\theta^{BS}$. Finally, the retailer decides on a retail price $p^{BS}$ to maximize her profit. Therefore, the retailer-led both-sharing game problem in Strategy BS can be given by:

(32) $$\left\{\begin{array}{c} \underset{\beta^{BS},\varphi^{BS}}{max}\pi_{r}^{BS}\left(\beta^{BS},\varphi^{BS}\right)=\left(\left(1-\varphi^{BS}\right)p^{BS*}-w^{BS*}\right)q^{BS}-\frac{1}{2}\beta^{BS}k\theta^{BS*}{}^{2}\\ w^{BS*}\;and\;\theta^{BS*}\;are\;derived\;from\;solving\;the\;following\;problem\\ \left\{\begin{array}{c} \underset{w^{BS},\theta^{BS}}{max}\begin{array}{c} \pi_{m}^{BS}\left(w^{BS},\theta^{BS}\right)=\left(\varphi^{BS}p^{BS*}+w^{BS}-c\right)q^{BS}-h\left(e\left(1-\theta^{BS}\right)q^{BS}-G\right)\\ -\frac{1}{2}\left(1-\beta^{BS}\right)k\theta^{BS}{}^{2} \end{array}\\ p^{BS*}\;is\;derived\;from\;solving\;the\;following\;problem\\ \underset{p^{BS}}{max}\pi_{r}^{BS}\left(p^{BS}\right)=\left(\left(1-\varphi^{BS}\right)p^{BS}-w^{BS}\right)q^{BS}-\frac{1}{2}\beta^{BS}k\theta^{BS}{}^{2} \end{array}\right. \end{array}\right.$$

where $q^{BS}$ is given by Equation (1). Solving the above problem, we can derive the following Theorem 4.

**Theorem** **4.** *In Strategy BS, the equilibrium cost-sharing and revenue-sharing percentages satisfy the relationship of* $\beta^{BS*}=$ *0 and* $\varphi^{BS*}=\frac{{\left(eh+\lambda \right)}^{2}}{2k}$ *, which implies that Strategy BS is equivalent to Strategy RS. The equilibrium solutions in Strategy BS are the same as those in Strategy RS.*

Theorem 4 indicates that when the retailer has both options of bearing CEA investment cost and sharing her revenue with the manufacturer, the retailer will always choose to share her revenue, while bearing the CEA investment cost is never a good choice. That is to say, Strategy BS is equivalent to Strategy RS. This finding is contrary to our intuition. This is because the retailer is better off in Strategy RS than in Strategy CS (refer to Proposition 1 below), as she would just share the revenue with her manufacturer rather than sharing the CEA investment cost simultaneously. That is to say, the retailer will give up the cost-sharing option, even if it is also available. Therefore, the equilibrium solutions in Strategy BS are identical to those in Strategy RS. Next, we will compare the equilibrium outcomes in the above four strategies in Proposition 1.

**Proposition** **1.** *The equilibrium CEA levels, order quantities, and profits under different incentive strategies satisfy*:

 *(i)*  $\theta^{BS*}=\theta^{RS*}>\theta^{CS*}>\theta^{PO*}$;
 *(ii)*  $q^{BS*}=q^{RS*}>q^{CS*}>q^{PO*}$;
 *(iii)*  $\pi_{r}^{BS*}=\pi_{r}^{RS*}>\pi_{r}^{CS*}>\pi_{r}^{PO*}$.

Proposition 1 reveals that when compared with Strategy PO, Strategies CS, RS, and BS strengthen the manufacturer’s incentive for carbon emission abatement, and Strategies RS and BS enhance the manufacturer’s motivation over Strategies CS. Moreover, in Strategies RS and BS, the retailer is inclined to place a larger order with the manufacturer than in Strategies CS and PO. Proposition 1 also demonstrates that the CEA level, order quantity, and retailer’s profit increase with the cooperation level. Therefore, for the retailer, it is the best choice to select Strategy RS (Strategy BS is equivalent to Strategy RS) to motivate the manufacturer to invest in CEA technology.

Next, our problem naturally appears: can Strategies RS and BS incentivize the manufacturer for abatement at the best level? In other words, can Strategies RS and BS achieve the same CEA level and supply chain profit as in a centralized supply chain? If not, how should we coordinate the low-carbon supply chain? Section 5 solves this problem.

## 5. Full Channel Coordination

In this section, we explore the equilibrium decisions and profit when a supply chain functions in full channel coordination. We find that the supply chain system is not coordinated under the above four strategies. Then, we propose a two-part tariff contract to coordinate the low-carbon supply chain.

### 5.1. Centralized Supply Chain

In the centralized supply chain model, the manufacturer and retailer act as one company. This model can be seen as one in which the manufacturer and the retailer are fully cooperative. In the centralized supply chain system, the manufacturer and retailer jointly decide on their retail price and CEA level. Thus, the optimization problem of the low-carbon supply chain can be formulated as:

(33) $$\underset{p^{C},\theta^{C}}{max}\pi_{sc}^{C}\left(p^{C},\theta^{C}\right)=\left(p^{C}-c\right)q^{C}-h\left(e\left(1-\theta^{C}\right)q^{C}-G\right)-\frac{1}{2}k\theta^{C}{}^{2},$$

where $q^{C}$ is given by Equation (1). Solving the above problem, we can derive the following Theorem 5.

**Theorem** **5.**  In the centralized supply chain system, the equilibrium pricing, order quantity, and CEA level of the supply chain are given by:

(34) $$p^{C*}=\frac{a\left(k-eh\left(eh+\lambda \right)\right)+\left(c+eh\right)\left(k-\lambda \left(eh+\lambda \right)\right)}{2k-{\left(eh+\lambda \right)}^{2}},$$

(35) $$q^{C*}=\frac{k\left(a-c-eh\right)}{2k-{\left(eh+\lambda \right)}^{2}},\;$$

*and*

(36) $$\theta^{C*}=\frac{\left(a-c-eh\right)\left(eh+\lambda \right)}{2k-{\left(eh+\lambda \right)}^{2}}.$$

Substituting the above equilibrium solutions given by Theorem 5 into Equation (33), we can derive the profits of the total supply chain:

(37) $$\pi_{sc}^{C*}=Gh+\frac{k{\left(a-c-eh\right)}^{2}}{2\left(2k-{\left(eh+\lambda \right)}^{2}\right)}.$$

Comparing the equilibrium decisions and total profits in the centralized supply chain and those in Strategies PO, CS, RS, and BS, we find that the supply chain cannot be fully coordinated by any of the above four incentive strategies. Therefore, we propose a two-part tariff contract to coordinate the low-carbon supply chain, which is widely used in supply chain management [52,53,54].

### 5.2. Two-Part Tariff Contract

We use superscript “TT” to denote the two-part tariff contract. Assume that the manufacturer provides a two-part tariff contract, $\left\{w^{TT},T\right\}$, where $w^{TT}$ denotes the wholesale price of the low-carbon products, and T is a fixed fee. Under the two-part tariff contract, the manufacturer also decides his CEA level $\theta^{TT}$. Similar to the analysis in Section 4.2, the problem of the retailer can be formulated as:

(38) $$\underset{p^{TT}}{max}\pi_{r}^{TT}\left(p^{TT}\right)=\left(p^{TT}-w^{TT}\right)q^{TT}-T.$$

Considering that T is a given fixed fee, the retailer has the following optimal response to the contract terms and CEA level of the manufacturer:

(39) $$p^{TT*}=\frac{1}{2}\left(a+w+\lambda \theta \right).$$

Given the retailer’s optimal response, the manufacturer tries to optimize his decisions by solving the following problem:

(40) $$\underset{w^{TT},\theta^{TT},T}{max}\pi_{m}^{TT}\left(w^{TT},\theta^{TT},T\right)=\left(w^{TT}-c\right)q^{TT}-h\left(e\left(1-\theta^{TT}\right)q^{TT}-G\right)-\frac{1}{2}k\theta^{TT}{}^{2}+T\;s.t.\;\left(p^{TT*}-w^{TT}\right)q^{TT}-T\geq \pi_{r}^{RS*}$$

where $p^{TT*}$ is given in Equation (39). Equation (40) is an incentive constraint that ensures that the retailer accepts the coordination contract provided by the manufacturer. Here, differently from related research, e.g., Hong and Guo [44], we assume that the retailer will only accept the two-part tariff contract if she earns at least the same profit as in Strategy RS but not in Strategy PO. This is because if the retailer cannot make so much profit, she will reject the contract and choose Strategy RS instead. The following Theorem 6 gives the manufacturer’s optimal decisions of the low-carbon supply chain under the two-part tariff contract.

**Theorem** **6.** *The manufacturer can coordinate the supply chain by offering the following two-part tariff contract and choosing the CEA level*$\theta^{TT*}=\theta^{C*}$:

(41) $$\left\{w^{TT*},T^{*}\right\}=\left\{\frac{\left(c+eh\right)\left(2k-\lambda \left(eh+\lambda \right)\right)-aeh\left(eh+\lambda \right)}{2k-{\left(eh+\lambda \right)}^{2}},\frac{k{\left(a-c-eh\right)}^{2}\left(6k+{\left(eh+\lambda \right)}^{2}\right)}{8{\left(2k-{\left(eh+\lambda \right)}^{2}\right)}^{2}}\right\}.$$

According to Theorem 6, under the above two-part tariff contract, the low-carbon supply chain can be fully coordinated and serves as a centralized supply chain where $\theta^{TT*}=\theta^{C*}$, $p^{TT*}=p^{C*},$ and $\pi_{sc}^{TT*}=\pi_{sc}^{C*}$. The retailer can achieve the same profit as in Strategy RS, and the manufacturer takes all the increased profit because he initiates the coordination contract and thus has more substantial bargaining power than the retailer.

## 6. Comparisons and Analyses

In the previous sections, we endeavor to drive the equilibrium decisions of the low-carbon supply chain under cap-and-trade regulation with different cooperation levels (i.e., Strategies PO, CS, RS, and BS). We also propose a two-part tariff contract to coordinate the low-carbon supply chain. In this section, we will investigate the impacts of incentive strategies on the manufacturer’s carbon emission abatement and explore the two firms’ incentive strategy preference and thus their equilibrium strategy choice. Furthermore, we analyze the environmental and social impacts of the four incentive strategies and the coordination contract.

### 6.1. Impact of Incentive Strategies on the Manufacturer’s Carbon Emission Abatement

To uncover the impact of different incentive strategies (i.e., Strategies PO, CS, RS, and BS) on the manufacturer’s incentive for abatement, we compare the optimal solutions under four incentive strategies with the best level (the centralized case), as the following Proposition 2 discloses.

**Proposition** **2.** *The optimal CEA levels, order quantities, wholesale prices, and retail prices under different incentive strategies satisfy:*

 *(i)* 
$\theta^{TT*}=\theta^{C*}>\theta^{BS*}=\theta^{RS*}>\theta^{CS*}>\theta^{PO*}$ *;*
 *(ii)* 
$q^{TT*}=q^{C*}>q^{BS*}=q^{RS*}>q^{CS*}>q^{PO*}$ *;*
 *(iii)* 
$w^{RS*}<w^{CS*},w^{PO*}$ *;*
 *(iv)*  $p^{TT*}=p^{C*}<p^{BS*}=p^{RS*}<p^{CS*},p^{PO*}$.

Proposition 2 suggests that when compared with Strategy PO, both Strategy RS and Strategy CS heighten the manufacturer’s incentive for carbon emission abatement, and Strategy RS improves the manufacturer’s motivation over Strategy CS but fails to induce the manufacturer to reduce emissions at the best level (i.e., that of the centralized system). This is because that the retailer’s sharing of CEA investment cost and retail revenue benefits the manufacturer and motivate him to choose a significant CEA level, which also increases the market demand and promote the retailer to place a larger order ($q^{RS*},q^{CS*}>q^{PO*}$). Furthermore, in Strategy RS, since the manufacturer can share the retailer retail earning, he will set a lower wholesale price than in Strategy CS ($w^{RS*}<w^{CS*}$), which also alleviates the double marginalization effect and prompts his retailer to order more and sell at a lower retail price ($q^{RS*}>q^{CS*},\;p^{RS*}<p^{CS*}$). In turn, the manufacturer can invest more in abatement technology and increase his CEA level in Strategy RS.

In addition, Strategy BS is equivalent to Strategy RS; hence the equilibrium decisions in Strategy BS are identical to those in Strategy RS. Furthermore, due to the double marginalization effect, the retail prices under the four strategies are higher than that in the centralized supply chain, while the CEA levels are lower than that in the centralized system. However, our proposed two-part tariff contract can coordinate the supply chain and reach the best-level optimal decisions.

**Figure 2:**
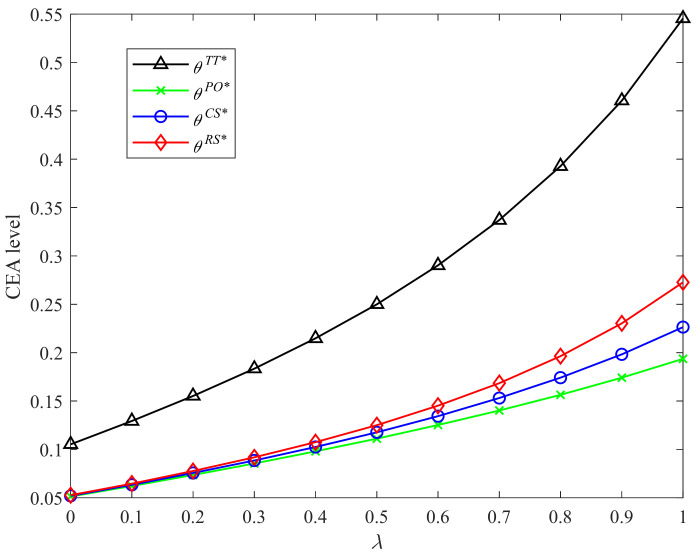
Comparison of CEA levels with different λ.

**Figure 3:**
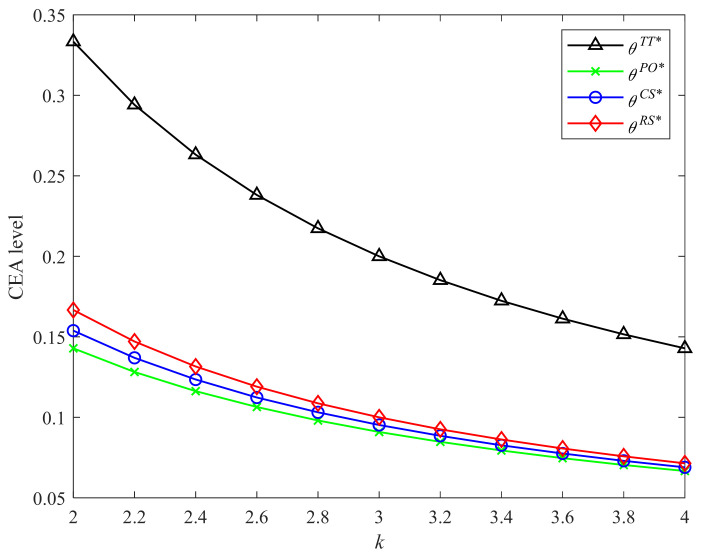
Comparison of CEA levels with different k.

Figure 2 and Figure 3 display graphically the comparison results of CEA levels in Strategies PO, CS, RS, and the two-part tariff contract with different CLA levels and CEA investment cost coefficients, respectively. It is easy to observe that the two-part tariff coordination contract and Strategy RS bring higher CEA levels than the other two strategies. The CEA level is highest in the two-part tariff contract. Strategy CS improves the manufacturer’s incentive for emissions abatement over Strategy PO. The CEA levels under four incentive strategies and two-part tariff contract increase in the CLA level and decrease in the CEA investment cost coefficient. The results shown in the two figures are consistent with Proposition 2 and Corollaries 1, 2, and 4.

### 6.2. Equilibrium Incentive Strategy Preference and Choice

Using the equilibrium solutions under four incentive strategies (i.e., Strategies PO, CS, RS, and BS) derived in the preceding sections, we explore how strategies types influence the firms’ profitability, and thus their equilibrium strategy choice. The results are shown below.

**Proposition** **3.** *The optimal profits of the manufacturer, retailer, and whole supply chain under different incentive strategies satisfy*:

 *(i)* 
$\pi_{m}^{TT*}>\pi_{m}^{BS*}=\pi_{m}^{RS*}>\pi_{m}^{CS*}>\pi_{m}^{PO*}$ *;*
 *(ii)* 
$\pi_{r}^{TT*}=\pi_{r}^{BS*}=\pi_{r}^{RS*}>\pi_{r}^{CS*}>\pi_{r}^{PO*}$ *;*
 *(iii)*  $\pi_{sc}^{TT*}=\pi_{sc}^{C*}>\pi_{sc}^{BS*}=\pi_{sc}^{RS*}>\pi_{sc}^{CS*}>\pi_{sc}^{PO*}$.

As illustrated by Proposition 3, when compared with Strategy PO, both the retailer and the manufacturer acquire more profits in Strategies RS and CS, and both firms gain more profits in Strategy RS than in Strategy CS. This result shows that both firms prefer Strategy CS to Strategy PO, prefer Strategy RS to Strategy CS, and they are indifferent between Strategy RS and Strategy BS. This finding is contrary to Li et al. [45], in which the retailer and manufacturer’s profits are both higher in Strategy CS than in Strategy RS when the marketing effort effect is relatively high. Similarly, in Strategy RS, the profit of the entire supply chain is higher than that in Strategy CS or Strategy PO but lower than that in the centralized system. In conclusion, without considering channel coordination, Strategy RS (equivalent to Strategy BS) is the equilibrium strategy for both firms. Considering the channel coordination, the two-part tariff contract is the equilibrium strategy/contract for both firms. We can also derive the managerial implication that with the increase of cooperation level, the profits of both firms are increasing. Therefore, both sides hope to have a higher level of cooperation. This managerial finding complements the study of Li et al. [45].

**Figure 4:**
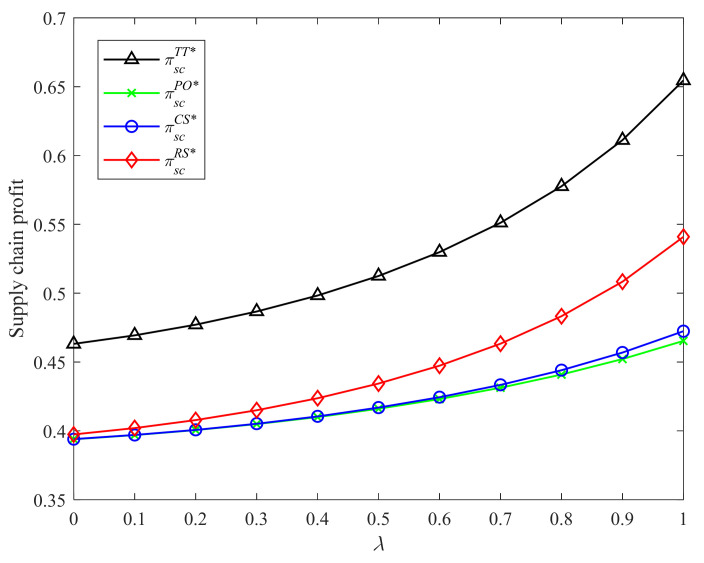
Comparison of the supply chain profits with different λ.

**Figure 5:**
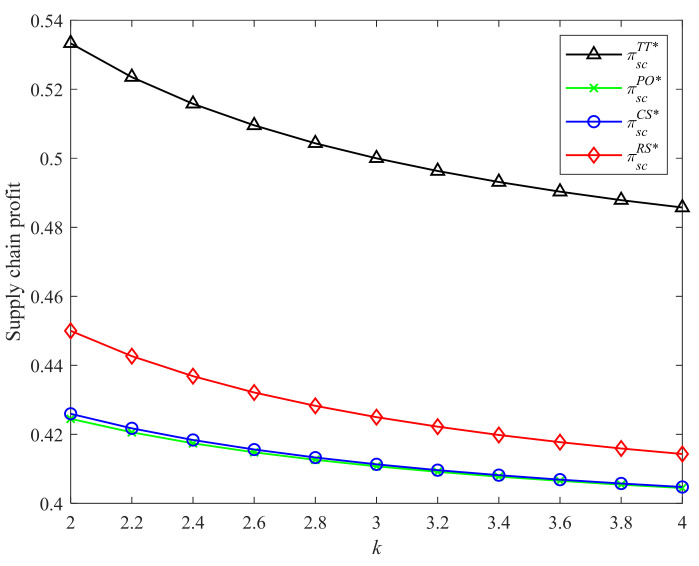
Comparison of the supply chain profits with different k.

Figure 4 and Figure 5 demonstrate the comparison results of supply chain profits in Strategies PO, CS, RS, and the two-part tariff contract with different CLA levels and CEA investment cost coefficients. It is easy to observe that the two-part tariff coordination contract and Strategy RS bring higher supply chain profits than the other two strategies. In addition, the supply chain profits under the four incentive strategies and two-part tariff contract increase in the CLA level and decrease in the CEA investment cost coefficient. The result is consistent with Proposition 3 and Corollaries 1, 2, and 4.

### 6.3. Environmental and Social Impacts of Different Incentive Strategies

In addition to the analyses of the economic benefits of the firms under different incentive strategies, we try to consider the environmental and social impacts of the four different incentive strategies (Strategies PO, CS, RS, and BS). Due to the complexity, the numerical analysis method is used to explore the comprehensive environmental and social impacts of four different incentive strategies. We assume that the model parameters satisfy: a=2, b=1,c=0.5, e=1, h=0.5, G=0.4, k=2.5,τ=0.9. With the above parameters’ combination, our models are solvable and our analysis is effective.

#### 6.3.1. Analysis of Total Carbon Emissions of Different Incentive Strategies

Because the manufacturer’s carbon emissions are harmful to the environment, we use the total carbon emissions to measure the negative environmental impact of different incentive strategies. The total carbon emissions of the manufacturer can be described as:

(42) $$E^{j*}=e\left(1-\theta^{j*}\right)q^{j*},$$

where $j\in \left\{PO,\;CS,\;RS,BS,TT,C\right\}$, $\theta^{j*},$ and $q^{j*}$ are the equilibrium CEA level and order quantity in Strategy j, respectively. Substituting the equilibrium solutions given by Theorems 1–5 into above Equation (42), we derive the total carbon emissions under four incentive strategies and the two-part tariff contract, which are as follows:

(43) $$E^{PO*}=\frac{ek\left(a-c-eh\right)\left(4k-\left(a-c+\lambda \right)\left(eh+\lambda \right)\right)}{{\left(4k-{\left(eh+\lambda \right)}^{2}\right)}^{2}},$$

(44) $$E^{CS*}=\frac{e\left(a-c-eh\right)\left(8k-{\left(eh+\lambda \right)}^{2}\right)\left(8k-\left(eh+\lambda \right)\left(2a-2c+eh+3\lambda \right)\right)}{4{\left(8k-3{\left(eh+\lambda \right)}^{2}\right)}^{2}},$$

(45) $$E^{BS*}=E^{RS*}=\frac{ek\left(a-c-eh\right)\left(4k-\left(eh+\lambda \right)\left(a-c+eh+2\lambda \right)\right)}{4{\left(2k-{\left(eh+\lambda \right)}^{2}\right)}^{2}},$$

(46) $$E^{TT*}=E^{C*}=\frac{ek\left(a-c-eh\right)\left(2k-\left(a-c+\lambda \right)\left(eh+\lambda \right)\right)}{{\left(2k-{\left(eh+\lambda \right)}^{2}\right)}^{2}}.$$

**Figure 6:**
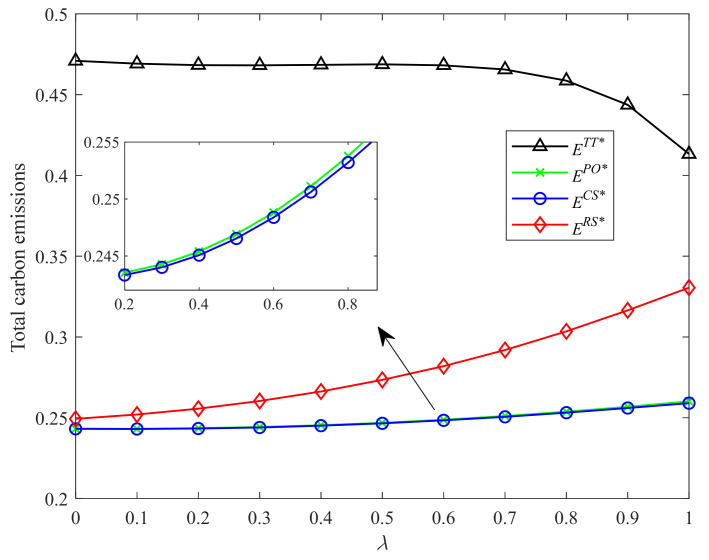
Comparison of total carbon emissions with different λ.

**Figure 7:**
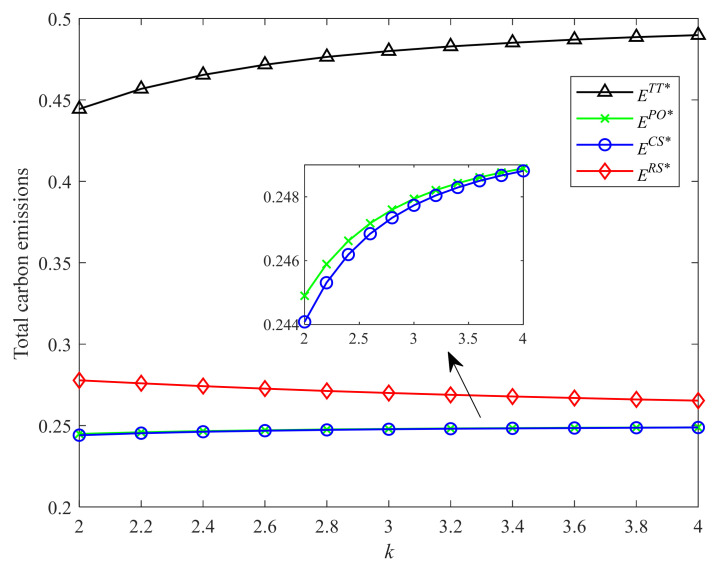
Comparison of total carbon emissions with different k.

Figure 6 and Figure 7 show the environmental impacts of different incentive strategies with different CLA levels and CEA investment cost coefficients, respectively. As shown in Figure 6, with the increase of the CLA level, the total carbon emissions in Strategies RS, CS, and PO increase. However, the carbon emissions under the two-part tariff contract are not always increasing in the CLA level. When the CLA level is relatively more significant, the carbon emissions under the two-part tariff contract decrease in the CLA level. As shown in Figure 7, with the increase of the CEA investment cost coefficient, i.e., the CEA technology being more expensive, the total carbon emissions in Strategies CS, PO, and two-part tariff contract increase. This result is intuitive. However, the carbon emissions in Strategy RS decrease in the CEA investment cost coefficient.

Moreover, the two-part tariff contract generates the most carbon emissions, followed by Strategy RS. This result shows that for Strategy RS and the two-part tariff contract, high profits are often accompanied by high carbon emissions. However, this conclusion is the opposite for Strategies CS and PO. Strategy CS brings higher profit but produces fewer carbon emissions than Strategy PO.

#### 6.3.2. Analysis of Eco-Social Welfare under Different Incentive Strategies

To measure the comprehensive environmental and social impacts of four different incentive strategies, we define the concept of eco-social welfare to denote the adverse environmental effects in social welfare. The eco-social welfare consists of three parts: profit of the entire supply chain, consumer surplus, and the negative environmental impact from carbon emissions. Hence, eco-social welfare is formulated as:

(47) $$ecoSW^{j*}=\pi_{sc}^{j*}+CS^{j*}-\tau E^{j*},$$

where the second term is consumer surplus in Strategy j, and τ is a weight that denotes how much environmental impact is valued compared with monetary welfare [55,56]. Consumer surplus measures a consumer’s additional benefit. It equals the maximum acceptable retail price minus the actual price. The consumer surplus can be given by:

(48) $$CS^{j*}=\int_{0}^{q^{j*}}\left(a+\lambda \theta^{j*}-x-p^{j*}\right)dx,$$

where $p^{j*},$ $q^{j*},$ and $\theta^{j*}$ are the equilibrium retail price, order quantity, and CEA level in Strategy j. Substituting the equilibrium solutions given by Theorems 1–5 into Equation (47), we obtain the eco-social welfare under four incentive strategies and the two-part tariff contract, which are as follows:

(49) $$ecoSW^{PO*}=\frac{{\left(a-c-eh\right)}^{2}k\left(7k-{\left(eh+\lambda \right)}^{2}\right)+2Gh{\left(4k-{\left(eh+\lambda \right)}^{2}\right)}^{2}-2\tau ek\left(a-c-eh\right)\left(4k-\left(a-c+\lambda \right)\left(eh+\lambda \right)\right)}{2{\left(4k-{\left(eh+\lambda \right)}^{2}\right)}^{2}},$$

(50) $$ecoSW^{CS*}=\frac{1}{48}\left(a^{2}-2ac+c^{2}-2aeh+2ceh+48Gh+e^{2}h^{2}\right)\;+\frac{\begin{array}{c} \left(a-c-eh\right)(128k\left(a-c-eh\right)\left(8k-3{\left(eh+\lambda \right)}^{2}\right)+3\left(a-c-eh\right){\left(8k-{\left(eh+\lambda \right)}^{2}\right)}^{2}\\ -24\tau e\left(8k-{\left(eh+\lambda \right)}^{2}\right)\left(8k-\left(eh+\lambda \right)\left(2a-2c+eh+3\lambda \right)\right)) \end{array}}{96{\left(8k-3{\left(eh+\lambda \right)}^{2}\right)}^{2}},$$

(51) $$ecoSW^{BS*}=ecoSW^{RS*}=Gh+\frac{k\left(a-c-eh\right)\left(\left(a-c-eh\right)\left(7k-3{\left(eh+\lambda \right)}^{2}\right)-2\tau e\left(4k-\left(eh+\lambda \right)\left(a-c+eh+2\lambda \right)\right)\right)}{8{\left(2k-{\left(eh+\lambda \right)}^{2}\right)}^{2}},$$

(52) $$ecoSW^{TT*}=ecoSW^{C*}=Gh+\frac{\left(a-c-eh\right)k\left(\left(a-c-eh\right)\left(3k-{\left(eh+\lambda \right)}^{2}\right)-2\tau e\left(2k-\left(a-c+\lambda \right)\left(eh+\lambda \right)\right)\right)}{2{\left(2k-{\left(eh+\lambda \right)}^{2}\right)}^{2}}.$$

**Figure 8:**
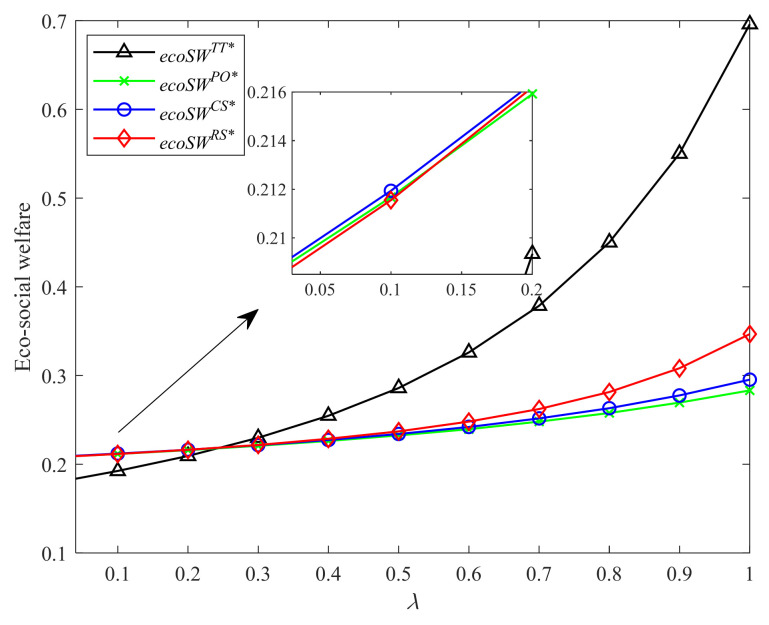
Comparison of eco-social welfare with different λ.

**Figure 9:**
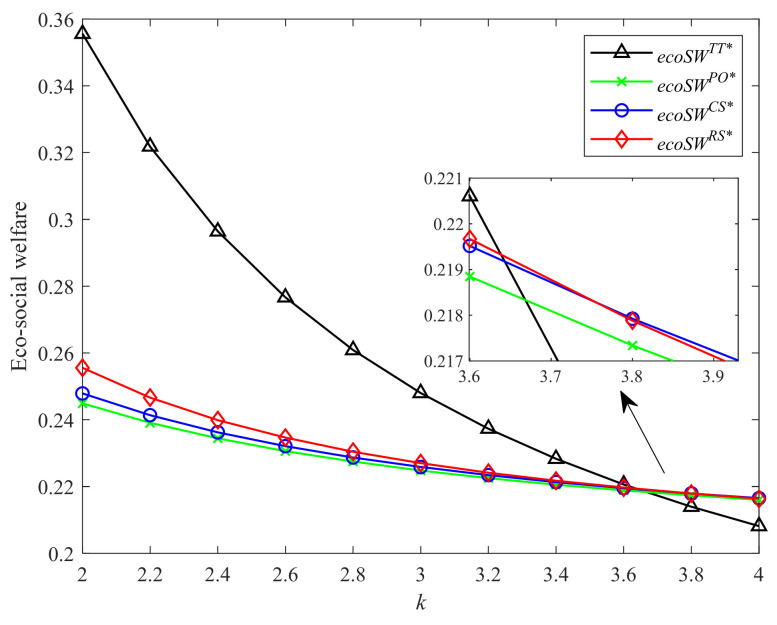
Comparison of eco-social welfare with different k.

Figure 8 and Figure 9 show the comprehensive impacts of four different incentive strategies on the environment and society with different CLA levels and CEA investment cost coefficients, respectively. As shown in Figure 8, the eco-social welfare under four incentive strategies increases the CLA level. When the CLA level is relatively large, the two-part tariff contract generates the most eco-social welfare, followed by Strategies RS, CS, and PO in sequence. This finding shows that the strategy with a higher profit produces more eco-social welfare. However, when the CLA level is relatively small, Strategy CS produces the most eco-social welfare. The two-part tariff contract brings the least eco-social welfare. This finding is contrary to the conclusion of Hong and Guo [44], in which they find that the coordination contract brings the most social welfare, followed by CS and PO contracts. Our result shows that when the consumers have lower low-carbon awareness, the higher cooperation level does not always produce higher eco-social welfare. As shown in Figure 9, the eco-social welfare in Strategies CS, PO, and two-part tariff contract decreases in CEA investment cost coefficient. When the CEA technology is not so much expensive, the higher cooperation level can often bring higher eco-social welfare. However, when the CEA investment becomes more costly, the high investment cost of CEA reduces the advantage of Strategy RS and the two-part tariff contract, which is also different from the findings of Hong and Guo [44]. Strategy CS brings more eco-social welfare than the other strategies under certain conditions.

## 7. Conclusions

In this study, we explore the incentive mechanisms in a two-echelon low-carbon supply chain, where consumers exhibit low-carbon awareness and market demand depends on the products’ low-carbon level under cap-and-trade regulation. To incentivize the manufacturer to invest in carbon emission abatement technology, the retailer can provide four strategies for the manufacturer, i.e., price-only, cost-sharing, revenue-sharing, and both-sharing strategies. The equilibrium decisions under four incentive strategies are obtained by establishing and solving game models. Moreover, we propose a two-part tariff contract to coordinate the low-carbon supply chain. At last, by comparisons and analyses, we investigate the impacts of four incentive strategies and coordination contract on the manufacturer’s carbon emission abatement behavior. Both firms’ incentive strategy preference and their equilibrium strategy choice are given. Furthermore, we analyze the environmental and social impacts of four incentive strategies and the two-part tariff contract. To sum up, we obtain the following theoretical results and managerial insights through theoretical research and analysis.

(1) In Strategies PO, CS, RS, and BS, the carbon quota given by the government has no impact on the equilibrium decisions of the low-carbon supply chain. A larger carbon quota can bring the manufacturer more profits while not affecting the retailer’s profit, which is similar to the findings of Xue and Sun [9]. Consumers’ higher low-carbon awareness can promote the manufacturer to invest more in CEA technology, thus increasing the manufacturer’s CEA level and the supply chain profit. If the CEA investment is expensive, the manufacturer is more inclined to buy extra carbon emission credits from the carbon trading market rather than invest in CEA technology. This gives us the vital management implication that cultivating consumers’ environmental awareness or improving CEA investment efficiency is conducive to the sustainable development of the low-carbon supply chain.
(2) In both Strategies CS and RS, if the consumers exhibit higher low-carbon awareness or CEA technology is not so expensive, the retailer will share a bigger proportion of CEA investment cost and her sales revenue to incentivize her manufacturer for abatement. The cooperation level between the retailer and manufacturer in Strategy RS is higher than that in Strategy CS. That is to say, the retailer is willing to share a higher percentage of sales revenue in Strategy RS relative to the proportion of CEA investment cost borne in Strategy CS. It is more effective for the retailer to share her revenue to incentivize the manufacturer than to bear the investment cost of CEA. Our findings complement the research of Hong and Guo [44], and Li et al. [45]. Thus, Strategy BS is equivalent to Strategy RS. The equilibrium solutions in Strategy BS are identical to those in Strategy RS.
(3) We propose a two-part tariff contract to coordinate the low-carbon supply chain, which is not investigated in Yang and Chen [49]. The low-carbon supply chain can be fully coordinated with the two-part tariff contract, where the optimal decisions can reach the best level. With the increase of cooperation level among the manufacturer and retailer, the manufacturer is becoming more willing to invest in CEA technology, thus increasing the profits of the supply chain members. Without channel coordination, Strategy RS (equivalent to Strategy BS) is the equilibrium strategy for both manufacturer and retailer. Considering the channel coordination, the two-part tariff contract is the equilibrium contract for both firms. To sum up, both firms hope to have a higher cooperation level.
(4) Considering the environmental and social impacts, high profits are often accompanied by high carbon emissions under four incentive strategies and the two-part tariff contract. When the consumers exhibit higher low-carbon awareness (or CEA technology is not so expensive), the strategy which brings higher profit always generates more eco-social welfare at the same time. In other words, a higher cooperation level can bring higher ecological social welfare. However, when the consumers exhibit lower low-carbon awareness or CEA technology is more expensive, a higher cooperation level cannot bring higher eco-social welfare. Moreover, Strategy CS brings more eco-social welfare than Strategies PO, RS, BS, and the two-part tariff contract under certain conditions.

Our paper provides important managerial insights and a decision-making reference for firms to establish appropriate cooperation mechanisms to promote sustainable development. However, there are still some limitations, leaving room for future research. For example, in this study, we only consider the vertical cooperation strategy within the supply chain. In the future, we can consider the horizontal cooperation strategy between different retailers and explore its influence on carbon emission abatement. Furthermore, the government has other emissions reduction policies, such as carbon tax policy and clean development mechanism [49,57]. Then, we can explore the incentive mechanisms for emissions abatement under similar carbon policies. Furthermore, in the platform economy era [58], the firm usually has multiple sales channels. Thus, it will also be interesting to explore the impacts of multiple channels cooperation on carbon emission abatement behavior in the future.

## Figures and Tables

**Table 1:** Main differences between our work and the relevant research.

Articles	Strategy Type			Carbon Policy		Coordination Issue	Focus Point
	PO	CS	RS	Cap-and-Trade	Carbon Tax		
Ghosh and Shah [43]		√				√	P
Qian et al. [50]	√			√		√	P, E
Wang et al. [41]	√	√		√			P
Yang and Chen [49]		√	√		√		P, E
Hong and Guo [44]	√	√				√	P, SW
Li et al. [45]	√	√	√				P
Our paper	√	√	√	√		√	P, SW, E

PO: price-only; CS: cost-sharing; RS: revenue-sharing; P: profit; SW: social welfare; E: environment.

**Table 2:** Symbols and notations used in this paper.

Notations	Descriptions
a	Initial market demand potential for products
q	Market demand for low-carbon products
λ	Demand sensitivity coefficient concerning the products’ CEA level
k	Cost coefficient of CEA technology investment
c	Production cost of the manufacturer
e	Initial carbon emissions of unit product
τ	Impact coefficient of carbon emissions on social welfare
E	Carbon emissions of the manufacturer
G	Total carbon quota from the government
T	Carbon trading amount of the manufacturer
i	$i\in \left\{m,\;r,\;sc\right\}$ denote the manufacturer, retailer, supply chain, respectively
j	$j\in \left\{PO,\;CS,\;RS,BS,TT,C\right\}$ denote the price-only strategy, cost-sharing strategy, revenue-sharing strategy, both-sharing strategy, two-part tariff contract, and centralized supply chain system, respectively
$\pi_{i}^{j}$	Profit of *i* in Strategy *j*
$SW^{j}$	Social welfare in Strategy *j*
**Decision Variables**	
θ	Carbon emission abatement level of the manufacturer
β	Proportion of CEA investment cost shared by the retailer
φ	Proportion of the retailer’s revenue shared by the manufacturer
w	Wholesale price of low-carbon products
p	Retail price of low-carbon products

## Data Availability

Not applicable.

## Appendix A

 **Proof of Theorem 1.**  Firstly, we solve the retailer’s optimization problem. Solving the second derivative of $\pi_{r}^{PO}\left(p\right)$ with respect to p yields $\frac{\partial^{2}\pi_{r}^{PO}\left(p\right)}{\partial p^{2}}=-2<0$. Hence, $\pi_{r}^{PO}\left(p\right)$ is a concave function with respect to p. By the first-order condition (FOC), i.e., $\frac{\partial \pi_{r}^{PO}\left(p\right)}{\partial p}=0$, we derive the optimal response function of the retailer: $p^{PO}=\frac{a+w+\theta \lambda }{2}.$

Secondly, we solve the manufacturer’s optimization problem. Substitute $p^{PO}$ into the manufacturer’s profit function $\pi_{m}^{PO}\left(w,\theta \right)$. Using $H\left(\pi_{m}^{PO}\right)$ to denote the hessian matrix of $\pi_{m}^{PO}\left(w,\theta \right)$, we have:

$$\frac{\partial^{2}\pi_{m}^{PO}\left(w,\theta \right)}{\partial w^{2}}=-1<0,\;$$

$$det\left(H\left(\pi_{m}^{PO}\right)\right)=\left|\begin{array}{cc} \frac{\partial^{2}\pi_{m}^{PO}\left(w,\theta \right)}{\partial w^{2}} & \frac{\partial^{2}\pi_{m}^{PO}\left(w,\theta \right)}{\partial w\partial \theta }\\ \frac{\partial^{2}\pi_{m}^{PO}\left(w,\theta \right)}{\partial \theta \partial w} & \frac{\partial^{2}\pi_{m}^{PO}\left(w,\theta \right)}{\partial w^{2}} \end{array}\right|=\frac{1}{4}\left(4k-{\left(eh+\lambda \right)}^{2}\right).\;$$

Based on the condition of $k>{\left(eh+\lambda \right)}^{2}$, the inequation of $\frac{1}{4}\left(4k-{\left(eh+\lambda \right)}^{2}\right)>0$ holds. Hence, $H\left(\pi_{m}^{PO}\right)$ is negative definite. $\pi_{m}^{PO}\left(w,\theta \right)$ is jointly concave in w and θ. By FOCs, i.e., $\frac{\partial \pi_{m}^{PO}\left(w,\theta \right)}{\partial w}=\frac{\partial \pi_{m}^{PO}\left(w,\theta \right)}{\partial \theta }=0,$ we can derive the optimal wholesale price and CEA level of the manufacturer, which are as follows:

$$\left\{\begin{array}{c} w^{PO*}=\frac{2k\left(a+eh\right)-eh\left(a+\lambda \right)\left(eh+\lambda \right)}{4k-{\left(eh+\lambda \right)}^{2}}\\ \theta^{PO*}=\frac{\left(a-eh\right)\left(eh+\lambda \right)}{4k-{\left(eh+\lambda \right)}^{2}} \end{array}\right..\;$$

Substituting the above optimal decisions into the optimal response price $p^{PO}$ of the retailer, we can derive the equilibrium pricing of the retailer:

$$p^{PO*}=\frac{k\left(3a+eh\right)-eh\left(a+\lambda \right)\left(eh+\lambda \right)}{4k-{\left(eh+\lambda \right)}^{2}}.$$

Substituting the above optimal decisions of the supply chain system into $q^{PO}$, we obtain the $q^{PO*}=\frac{k\left(a-c-eh\right)}{4k-{\left(eh+\lambda \right)}^{2}}$. Therefore, Theorem 1 is proved. □

 **Proof of Corollary 1.** 

(i) $\frac{\partial \theta^{PO*}}{\partial \lambda }=\frac{\left(a-c-eh\right)\left(4k+{\left(eh+\lambda \right)}^{2}\right)}{{\left(4k-{\left(eh+\lambda \right)}^{2}\right)}^{2}}>0$, $\frac{\partial q^{PO*}}{\partial \lambda }=\frac{2k\left(a-c-eh\right)\left(eh+\lambda \right)}{{\left(4k-{\left(eh+\lambda \right)}^{2}\right)}^{2}}>0$, $\frac{\partial \pi_{m}^{PO*}}{\partial \lambda }=\frac{k{\left(a-c-eh\right)}^{2}\left(eh+\lambda \right)}{{\left(4k-{\left(eh+\lambda \right)}^{2}\right)}^{2}}>0$, $\frac{\partial \pi_{r}^{PO*}}{\partial \lambda }=\frac{4k^{2}{\left(a-c-eh\right)}^{2}\left(eh+\lambda \right)}{{\left(4k-{\left(eh+\lambda \right)}^{2}\right)}^{3}}>0$, $\frac{\partial \pi_{sc}^{PO*}}{\partial \lambda }=\frac{{\left(a-c-eh\right)}^{2}k\left(eh+\lambda \right)\left(8k-{\left(eh+\lambda \right)}^{2}\right)}{{\left(4k-{\left(eh+\lambda \right)}^{2}\right)}^{3}}>0$;
(ii) $\frac{\partial \theta^{PO*}}{\partial k}=-\frac{4\left(a-c-eh\right)\left(eh+\lambda \right)}{{\left(4k-{\left(eh+\lambda \right)}^{2}\right)}^{2}}<0$, $\frac{\partial q^{PO*}}{\partial k}=-\frac{\left(a-c-eh\right){\left(eh+\lambda \right)}^{2}}{{\left(4k-{\left(eh+\lambda \right)}^{2}\right)}^{2}}<0$, $\frac{\partial \pi_{m}^{PO*}}{\partial k}=-\frac{{\left(a-c-eh\right)}^{2}{\left(eh+\lambda \right)}^{2}}{2{\left(4k-{\left(eh+\lambda \right)}^{2}\right)}^{2}}<0$, $\frac{\partial \pi_{r}^{PO*}}{\partial k}=-\frac{2{\left(a-c-eh\right)}^{2}k{\left(eh+\lambda \right)}^{2}}{{\left(4k-{\left(eh+\lambda \right)}^{2}\right)}^{3}}<0$, $\frac{\partial \pi_{sc}^{PO*}}{\partial k}=-\frac{{\left(a-c-eh\right)}^{2}{\left(eh+\lambda \right)}^{2}\left(8k-{\left(eh+\lambda \right)}^{2}\right)}{2{\left(4k-{\left(eh+\lambda \right)}^{2}\right)}^{3}}<0$. □

 **Proof of Theorem 2.**  The solving procedures of the manufacturer and retailer’s optimization problems are similar to the proof of Theorem 1. Similarly, we can derive the optimal response functions of the manufacturer and retailer, which are as follows:

$$w^{CS}=\frac{2k\left(1-\beta \right)\left(a+eh\right)-eh\left(eh+\lambda \right)\left(a+\lambda \right)}{4k\left(1-\beta \right)-{\left(eh+\lambda \right)}^{2}},\;$$

$$\theta^{CS}=\frac{\left(a-eh\right)\left(eh+\lambda \right)}{4k\left(1-\beta \right)-{\left(eh+\lambda \right)}^{2}},\;$$

$$p^{CS}=\frac{k\left(1-\beta \right)\left(3a+eh\right)-eh\left(eh+\lambda \right)\left(a+\lambda \right)}{4k\left(1-\beta \right)-{\left(eh+\lambda \right)}^{2}}.\;$$

Substituting the above optimal response functions into the profit function of the retailer, we can derive $\pi_{r}^{CS}\left(\beta \right)=\frac{k{\left(a-eh\right)}^{2}\left(2k{\left(1-\beta \right)}^{2}-\beta {\left(eh+\lambda \right)}^{2}\right)}{2{\left(4k\left(1-\beta \right)-{\left(eh+\lambda \right)}^{2}\right)}^{2}}$. By the FOC, i.e., $\frac{\partial \pi_{r}^{CS}\left(\beta \right)}{\partial \beta }=0$, we derive the optimal cost-sharing ratio of the retailer: $\beta^{CS*}=\frac{{\left(eh+\lambda \right)}^{2}}{8k}.$ Substituting $\beta^{CS*}$ into the optimal response functions of the manufacturer and retailer, we can derive:

$$w^{CS^{*}}=\frac{8k\left(a+eh\right)-\left(eh+\lambda \right)\left(a\left(5eh+\lambda \right)+eh\left(eh+5\lambda \right)\right)}{2\left(8k-3{\left(eh+\lambda \right)}^{2}\right)},\;$$

$$p^{CS^{*}}=\frac{8k\left(3a+eh\right)-\left(eh+\lambda \right)\left(a\left(11eh+3\lambda \right)+eh\left(eh+9\lambda \right)\right)}{4\left(8k-3{\left(eh+\lambda \right)}^{2}\right)},\;$$

$$\theta^{CS^{*}}=\frac{2\left(a-eh\right)\left(eh+\lambda \right)}{8k-3{\left(eh+\lambda \right)}^{2}}.\;$$

Substituting the above optimal decisions of the supply chain into $q^{CS}$, we can obtain the optimal $q^{CS*}=\frac{\left(a-c-eh\right)\left(8k-{\left(eh+\lambda \right)}^{2}\right)}{4\left(8k-3{\left(eh+\lambda \right)}^{2}\right)}$. Therefore, Theorem 2 is proved. □

 **Proof of Corollary 2.**  The proof of Corollary 2 is similar to the proof of Corollary 1, so we omit it here. □

 **Proof of Theorem 3.**  The proof of Theorem 3 is similar to the proof of Theorem 2, so we omit it here. □

 **Proof of Corollary 3.**  The proof of Corollary 3 is easy to derive, so we omit it here. □

 **Proof of Corollary 4.**  The proof of Corollary 4 is similar to the proof of Corollary 1, so we omit it here. □

 **Proof of Theorem 4.**  The proof of Theorem 4 is similar to the proof of Theorem 2, so we omit it here. □

 **Proof of Theorem 5.**  We solve the supply chain’s optimization problem. Using $H\left(\pi_{sc}^{C}\right)$ to denote the hessian matrix of $\pi_{mi}^{TC}\left(w_{i},e_{i}\right)$, we have:

$$\frac{\partial^{2}\pi_{sc}^{C}\left(p,\theta \right)}{\partial p^{2}}=-2<0,\;$$

$$det\left(H\left(\pi_{sc}^{C}\right)\right)=\left|\begin{array}{cc} \frac{\partial^{2}\pi_{sc}^{C}\left(p,\theta \right)}{\partial p^{2}} & \frac{\partial^{2}\pi_{sc}^{C}\left(p,\theta \right)}{\partial p\partial \theta }\\ \frac{\partial^{2}\pi_{sc}^{C}\left(p,\theta \right)}{\partial p\partial \theta } & \frac{\partial^{2}\pi_{sc}^{C}\left(p,\theta \right)}{\partial \theta^{2}} \end{array}\right|=2k-{\left(eh+\lambda \right)}^{2}.\;$$

Based on the condition of $k>{\left(eh+\lambda \right)}^{2}$, hence, $H\left(\pi_{sc}^{C}\right)$ is negative definite. $\pi_{sc}^{C}\left(p,\theta \right)$ is jointly concave in p and θ. By FOCs, i.e., $\frac{\partial \pi_{sc}^{C}\left(p,\theta \right)}{\partial p}=\frac{\partial \pi_{sc}^{C}\left(p,\theta \right)}{\partial \theta }=0,$ we can derive the optimal retail price and CEA level of the supply chain system, which are as follows:

$$\left\{\begin{array}{c} p^{C*}=\frac{k\left(a+eh\right)-eh\left(a+\lambda \right)\left(eh+\lambda \right)}{2k-{\left(eh+\lambda \right)}^{2}}\\ \theta^{C*}=\frac{\left(a-eh\right)\left(eh+\lambda \right)}{2k-{\left(eh+\lambda \right)}^{2}} \end{array}\right..$$

Substituting the above optimal decisions of the supply chain into $q^{C}$, we obtain $q^{C*}=\frac{k\left(a-c-eh\right)}{2k-{\left(eh+\lambda \right)}^{2}}$. Therefore, Theorem 1 is proved. □

 **Proof of Proposition 1.**  It is easy to derive that (i) $\theta^{RS*}-\theta^{CS*}=\frac{\left(a-c-eh\right){\left(eh+\lambda \right)}^{3}}{2\left(2k-{\left(eh+\lambda \right)}^{2}\right)\left(8k-3{\left(eh+\lambda \right)}^{2}\right)}>0$, $\theta^{CS*}-\theta^{PO*}=\frac{\left(a-c-eh\right){\left(eh+\lambda \right)}^{3}}{\left(4k-{\left(eh+\lambda \right)}^{2}\right)\left(8k-3{\left(eh+\lambda \right)}^{2}\right)}>0$; (ii) $q^{RS*}-q^{CS*}=\frac{\left(a-c-eh\right){\left(eh+\lambda \right)}^{2}\left(4k-{\left(eh+\lambda \right)}^{2}\right)}{4\left(2k-{\left(eh+\lambda \right)}^{2}\right)\left(8k-3{\left(eh+\lambda \right)}^{2}\right)}>0$, $q^{CS*}-q^{PO*}=\frac{\left(a-c-eh\right){\left(eh+\lambda \right)}^{4}}{4\left(4k-{\left(eh+\lambda \right)}^{2}\right)\left(8k-3{\left(eh+\lambda \right)}^{2}\right)}>0$; (iii) $\pi_{r}^{RS*}-\pi_{r}^{CS*}=\frac{{\left(a-c-eh\right)}^{2}{\left(eh+\lambda \right)}^{4}}{16\left(2k-{\left(eh+\lambda \right)}^{2}\right)\left(8k-3{\left(eh+\lambda \right)}^{2}\right)}>0$, $\pi_{r}^{CS*}-\pi_{r}^{PO*}=\frac{{\left(a-c-eh\right)}^{2}{\left(eh+\lambda \right)}^{6}}{16{\left(4k-{\left(eh+\lambda \right)}^{2}\right)}^{2}\left(8k-3{\left(eh+\lambda \right)}^{2}\right)}>0$. Thus, we can derive (i) $\theta^{RS*}>\theta^{CS*}>\theta^{PO*}$; (ii) $q^{RS*}>q^{CS*}>q^{PO*}$; (iii) $\pi_{r}^{RS*}>\pi_{r}^{CS*}>\pi_{r}^{PO*}$. Because Strategy BS is equivalent to Strategy RS. Then Proposition 1 is proved. □

 **Proof of Theorem 6.**  Based on the optimization problem faced by the manufacturer, the manufacturer will extract all the surplus by setting the appropriate value of T from the retailer. That is to say, the manufacturer will choose $T^{*}=\left(p^{TT*}-w^{TT}\right)q^{TT}-\pi_{r}^{RS*}.$ Substituting it into the profit function of the manufacturer, thus his optimization problem is as follows:

$$\underset{w^{TT},\theta^{TT}}{max}\pi_{m}^{TT}\left(w^{TT},\theta^{TT},T\right)=\left(p^{TT*}-c\right)q^{TT}-h\left(e\left(1-\theta^{TT}\right)q^{TT}-G\right)-\frac{1}{2}k\theta^{TT}{}^{2}-\pi_{r}^{RS*}.\;$$

$p^{TT*}$ is given by Equation (39). Solving the above problem, we derive:

$$\left\{\begin{array}{c} w^{TT*}=\frac{\left(c+eh\right)\left(2k-\lambda \left(eh+\lambda \right)\right)-aeh\left(eh+\lambda \right)}{2k-{\left(eh+\lambda \right)}^{2}}\\ \theta^{TT*}=\frac{\left(a-c-eh\right)\left(eh+\lambda \right)}{2k-{\left(eh+\lambda \right)}^{2}} \end{array}\right..\;$$

Substituting the above optimal decisions of the manufacturer into Equation (39), we obtain:

$$p^{TT*}=\frac{a\left(k-eh\left(eh+\lambda \right)\right)+\left(c+eh\right)\left(k-\lambda \left(eh+\lambda \right)\right)}{2k-{\left(eh+\lambda \right)}^{2}}.\;$$

Substituting above $w^{TT*}$, $\theta^{TT*}$ and $p^{TT*}$ into $T^{*}=\left(p^{TT*}-w^{TT}\right)q^{TT}-\pi_{r}^{RS*}$, we derive:

$$T^{*}=\frac{k{\left(a-c-eh\right)}^{2}\left(6k+{\left(eh+\lambda \right)}^{2}\right)}{8{\left(2k-{\left(eh+\lambda \right)}^{2}\right)}^{2}}.\;$$

Therefore, in the two-part tariff contract, the optimal decisions of the manufacturer and retailer terms are derived. □

 **Proof of Proposition 2.**  

(i) It is easy to derive that $\theta^{C*}-\theta^{RS*}=\frac{\left(a-c-eh\right)\left(eh+\lambda \right)}{2\left(2k-{\left(eh+\lambda \right)}^{2}\right)}>0$, $\theta^{RS*}-\theta^{CS*}=\frac{\left(a-c-eh\right){\left(eh+\lambda \right)}^{3}}{2\left(2k-{\left(eh+\lambda \right)}^{2}\right)\left(8k-3{\left(eh+\lambda \right)}^{2}\right)}>0$, $\theta^{CS*}-\theta^{PO*}=\frac{\left(a-c-eh\right){\left(eh+\lambda \right)}^{3}}{\left(4k-{\left(eh+\lambda \right)}^{2}\right)\left(8k-3{\left(eh+\lambda \right)}^{2}\right)}>0$, thus, $\theta^{C*}>\theta^{RS*}>\theta^{CS*}>\theta^{PO*}$;
(ii) It is easy to drive that $q^{C*}-q^{RS*}=\frac{\left(a-c-eh\right)k}{2\left(2k-{\left(eh+\lambda \right)}^{2}\right)}>0$, $q^{RS*}-q^{CS*}=\frac{\left(a-c-eh\right){\left(eh+\lambda \right)}^{2}\left(4k-{\left(eh+\lambda \right)}^{2}\right)}{4\left(2k-{\left(eh+\lambda \right)}^{2}\right)\left(8k-3{\left(eh+\lambda \right)}^{2}\right)}>0$, $q^{CS*}-q^{PO*}=\frac{\left(a-c-eh\right){\left(eh+\lambda \right)}^{4}}{4\left(4k-{\left(eh+\lambda \right)}^{2}\right)\left(8k-3{\left(eh+\lambda \right)}^{2}\right)}>0$, thus, $q^{C^{*}}>q^{RS*}>q^{CS*}>q^{PO*}$;
(iii) It is easy to drive that $w^{RS*}-w^{CS*}=-\frac{{\left(eh+\lambda \right)}^{2}\left(\left(c+eh\right)\left(4k-3\lambda \left(eh+\lambda \right)\right)+3a\left(4k-\left(eh+\lambda \right)\left(2eh+\lambda \right)\right)\right)}{4k\left(8k-3{\left(eh+\lambda \right)}^{2}\right)}<0$, $w^{RS*}-w^{PO*}=-\frac{{\left(eh+\lambda \right)}^{2}\left(\left(c+eh\right)\left(2k-\lambda \left(eh+\lambda \right)\right)+a\left(6k-\left(eh+\lambda \right)\left(2eh+\lambda \right)\right)\right)}{4k\left(4k-{\left(eh+\lambda \right)}^{2}\right)}<0$, thus, $w^{RS*}<w^{CS*},w^{PO*}$;
(iv) It is easy to drive that $p^{C*}-p^{RS*}=-\frac{\left(a-c-eh\right)\left(k-\lambda \left(eh+\lambda \right)\right)}{2\left(2k-{\left(eh+\lambda \right)}^{2}\right)}<0$, $p^{RS*}-p^{CS*}=-\frac{\left(a-c-eh\right){\left(eh+\lambda \right)}^{2}\left(4k-\left(eh+\lambda \right)\left(eh+3\lambda \right)\right)}{4\left(2k-{\left(eh+\lambda \right)}^{2}\right)\left(8k-3{\left(eh+\lambda \right)}^{2}\right)}<0$, $p^{RS*}-p^{PO*}=-\frac{\left(a-c-eh\right){\left(eh+\lambda \right)}^{2}\left(k-\lambda \left(eh+\lambda \right)\right)}{2\left(2k-{\left(eh+\lambda \right)}^{2}\right)\left(4k-{\left(eh+\lambda \right)}^{2}\right)}<0$, thus, $p^{C*}<p^{RS*}<p^{CS*},p^{PO*}$.

Moreover, Strategy BS is equivalent to Strategy RS. The two-part tariff contract can coordinate the supply chain. Therefore, Proposition 2 is proved. □

 **Proof** **of** **Proposition** **3.** 

(i) It is easy to drive that $\pi_{m}^{RS*}-\pi_{m}^{CS*}=\frac{{\left(a-c-eh\right)}^{2}{\left(eh+\lambda \right)}^{2}\left(4k-{\left(eh+\lambda \right)}^{2}\right)}{8\left(2k-{\left(eh+\lambda \right)}^{2}\right)\left(8k-3{\left(eh+\lambda \right)}^{2}\right)}>0$, $\pi_{m}^{CS*}-\pi_{m}^{PO*}=\frac{{\left(a-c-eh\right)}^{2}{\left(eh+\lambda \right)}^{4}}{8\left(4k-{\left(eh+\lambda \right)}^{2}\right)\left(8k-3{\left(eh+\lambda \right)}^{2}\right)}>0$, thus, $\pi_{m}^{RS*}>\pi_{m}^{CS*}>\pi_{m}^{PO*}$;
(ii) It is easy to drive that $\pi_{r}^{RS*}-\pi_{r}^{CS*}=\frac{{\left(a-c-eh\right)}^{2}{\left(eh+\lambda \right)}^{4}}{16\left(2k-{\left(eh+\lambda \right)}^{2}\right)\left(8k-3{\left(eh+\lambda \right)}^{2}\right)}>0$, $\pi_{r}^{CS*}-\pi_{r}^{PO*}=\frac{{\left(a-c-eh\right)}^{2}{\left(eh+\lambda \right)}^{6}}{16{\left(4k-{\left(eh+\lambda \right)}^{2}\right)}^{2}\left(8k-3{\left(eh+\lambda \right)}^{2}\right)}>0$, thus, $\pi_{r}^{RS*}>\pi_{r}^{CS*}>\pi_{r}^{PO*}$;
(iii) It is easy to drive that $\pi_{sc}^{C*}-\pi_{sc}^{RS*}=\frac{{\left(a-c-eh\right)}^{2}k}{8\left(2k-{\left(eh+\lambda \right)}^{2}\right)}>0$, $\pi_{sc}^{RS*}-\pi_{sc}^{CS*}=\frac{{\left(a-c-eh\right)}^{2}{\left(eh+\lambda \right)}^{2}\left(8k-{\left(eh+\lambda \right)}^{2}\right)}{16\left(2k-{\left(eh+\lambda \right)}^{2}\right)\left(8k-3{\left(eh+\lambda \right)}^{2}\right)}>0$, $\pi_{sc}^{CS*}-\pi_{sc}^{PO*}=\frac{{\left(a-c-eh\right)}^{2}{\left(eh+\lambda \right)}^{4}\left(8k-{\left(eh+\lambda \right)}^{2}\right)}{16{\left(4k-{\left(eh+\lambda \right)}^{2}\right)}^{2}\left(8k-3{\left(eh+\lambda \right)}^{2}\right)}>0$, thus, $\pi_{sc}^{C*}>\pi_{sc}^{RS*}>\pi_{sc}^{CS*}>\pi_{sc}^{PO*}$.

Moreover, Strategy BS is equivalent to Strategy RS. Therefore, Proposition 3 is proved. □
